# An analytical study on the identification of N-linked glycosylation sites using machine learning model

**DOI:** 10.7717/peerj-cs.1069

**Published:** 2022-09-21

**Authors:** Muhammad Aizaz Akmal, Muhammad Awais Hassan, Shoaib Muhammad, Khaldoon S. Khurshid, Abdullah Mohamed

**Affiliations:** 1 Department of Computer Science, University of Engineering and Technology, KSK, Lahore, Punjab, Pakistan; 2 Department of Computer Science, University of Engineering and Technology, Lahore, Punjab, Pakistan; 3 Research Centre, Future University in Egypt, New Cairo, Egypt

**Keywords:** N-linked, Glycosylation, Machine learning, Deep learning, Artificial intelligence, Performance evaluation criteria

## Abstract

N-linked is the most common type of glycosylation which plays a significant role in identifying various diseases such as type I diabetes and cancer and helps in drug development. Most of the proteins cannot perform their biological and psychological functionalities without undergoing such modification. Therefore, it is essential to identify such sites by computational techniques because of experimental limitations. This study aims to analyze and synthesize the progress to discover N-linked places using machine learning methods. It also explores the performance of currently available tools to predict such sites. Almost seventy research articles published in recognized journals of the N-linked glycosylation field have shortlisted after the rigorous filtering process. The findings of the studies have been reported based on multiple aspects: publication channel, feature set construction method, training algorithm, and performance evaluation. Moreover, a literature survey has developed a taxonomy of N-linked sequence identification. Our study focuses on the performance evaluation criteria, and the importance of N-linked glycosylation motivates us to discover resources that use computational methods instead of the experimental method due to its limitations.

## Introduction

The process of glycosylation is considered to be one of the most complex type of post translation modification (PTM) in eukaryotes cells (Akmal, Rasool & Khan, 2017; Yang et al., 2019). The post translation modification occurs when protein, after synthesis, undergo different type of changes and without these modification proteins cannot perform their psychological functionalities properly (Yang et al., 2019). Nearly 200 different types of such post translation modification have been discovered and glycosylation is most important amongst them as it plays a vital role in different biological functions such as cell communication, protein folding, recognition of antigens and −50% of the human genomes are glycosylated (Akmal, Rasool & Khan, 2017; Akmal et al., 2020; Yang et al., 2019). The glycosylation sites are very relevant for cancer discovery as well as for further drug development (He, Wei & Zou, 2019; Hwang et al., 2020). Glycosylation sites are classified into five types: N-linked, O-linked, C-linked, glypiation and phospho glycosylation (Lei, Tang & Du, 2017). It is very much important to identify such sites.

There are various techniques to identify such sites, broadly it can be classified into experimental and computational method (Audagnotto & Dal Peraro, 2017). The experimental method requires the understanding of cell biology and the functions of cell structure (Hwang et al., 2020). The well-know techniques used for experimental identification are: radioactive label, chromatin immunoprecipitation (ChIP), mass spectrometry (MS) and liquid chromatography (LCG) (Akmal et al., 2020; Hwang et al., 2020; Naseer et al., 2020a). In computational method, researchers discover valuable information from the structure of protein sequences and apply some artificially intelligent algorithms to predict the relevant glycosylation or any other PTM sites (Hamby & Hirst, 2008; He, Wei & Zou, 2019; Shek, Kotidis & Betenbaugh, 2021; Naseer et al., 2021b; Murad et al., 2021).

The N-linked glycosylation is the primary glycosylation type, as 90% of glycosylated sites belong to the N-linked glycosylation (Akmal, Rasool & Khan, 2017). Usually, N-glycans are attached to glycoproteins on asparagine residues within the Asn-X-Ser/Thr sequon (except proline, X could be any amino residue) (Zhang et al., 2021b; Alkuhlani et al., 2021). N-linked glycans plays vital role in intrinsic and extrinsic (Alkuhlani et al., 2021). Apart from improving the protein’s stability, it provides a structural component to the cell surface. N-glycan also mediate cell-to-cell interaction and controls the glycoprotein in the cellular environment (Naseer et al., 2020b). N-linked glycan helps is identification of various diseases such as type I diabetes, cancer, rheumatoid arthritis, and Crohn’s disease (Alkuhlani et al., 2021; Naseer et al., 2020a; Khan et al., 2020b). Therefore, it is very much important to identify such sites, but the identification of such sites using experimental technique is time-consuming and expensive as well (Coff et al., 2020; Akmal et al., 2020; Qiu et al., 2018). Therefore, researchers have developed several computational models based on artificial neural network (ANN) to predict the N-linked sites (Le, Sandag & Ou, 2018; Butt et al., 2016; Alkuhlani et al., 2021). Although, few reviews exist on N-linked prediction model, but they mainly focus on algorithm used to train the model and less focused on the feature set construction and performance metric, as shown in Table 1. These studies only analyzed the models developed up to 2019.

**Table 1:** Proposed survey comparison with existing studies.

Article Ref. No.	Focus	Year	Survey approach	Quality assessment	N-linked model (Tool)	Feature construction	Training algorithm	Organism type	Performance metric (ACC, SN, SP)	Target repository
Alkuhlani et al. (2021)	Glycosylation sites prediction tool using AI.	2021	Informal	✗	✗	✓	✓	✓	✗	✗
Shek, Kotidis & Betenbaugh (2021)	Experimental and computation method for PTM site prediction	2021	Informal	✗	✗	✗	✓	✗	✗	✗
Kuo-Chen (2019)	PTM sites prediction model develop using Chou’s 5 step model.	2019	Informal	✗	✗ (other PTM)	✓	✓	✗	✗	✗
He, Wei & Zou (2019)	Research progress in PTM site prediction.	2019	Informal	✗	✗ (glyco type not specified)	✓	✓	✗	✗	✗
Audagnotto & Dal Peraro (2017)	Tools used for PTM.	2017	Informal	✗	✓	✗	✓	✗	✗	✗
This survey	N-linked site prediction tool including training algorithm, and feature approach which helps to construct an efficient model for other PTM.	2021	Systematic Review	✓	✓	✓	✓	✓	✓	5

The glycosylated region of N-linked sites appears at the specific location within the protein sequence, as protein sequence consists of the chain of amino acid and each amino acid out of known 20 is represented by specific alphabetic character (Qiu et al., 2018; Yang et al., 2019; Kumari, Kumar & Kumar, 2018). In computational approach, it is required to extract some useful information from these sequences to construct the feature vector (Butt, Rasool & Khan, 2017; Chien et al., 2020; Hamby & Hirst, 2008; Naseer et al., 2021b). The feature vectors of glycosylated and non-glycosylated N-linked sites have certain pattern of protein sequences and these patterns have identified through the various technique (algorithm) of machine learning method (Taherzadeh et al., 2019; Tran, Pham & Ou, 2021; Hayat & Khan, 2011; Park et al., 2019; Xiang, Zou & Zhao, 2021; Dimeglio et al., 2020). The evidence of organism type also helps in the successful identification of such sites (Huang & Li, 2018).

The existing reviews are compared on various perspectives such as quality assessment scores, availability of N-linked model, feature set construction method, training model algorithm, specie type, performance metric and target repositories as shown in Table 1. The proposed study only focused on the review articles accepted in recognized journals because of reliability (Barukab et al., 2019). This comparison helps the need to build the survey.

The rational of our work is to provide the comprehensive systematic literature review on the identification of N-linked sites to bring out the detail of exiting computational models. The researchers have performed numerous efforts to identify such sites computationally in the recent past. The work presented by these researchers has been reviewed by few authors to ensure the effectiveness of the proposed prediction model to identify the N-linked sites (Shek, Kotidis & Betenbaugh, 2021; Alkuhlani et al., 2021; Audagnotto & Dal Peraro, 2017). The authors primarily focused on the feature set construction algorithm and training algorithm, and less or no focus on quality assessment criteria, performance metric evaluation and the type of species of the reviewed articles used to predict the N-linked sites. The proposed systematic review provides novel features such as targeting channel, quality assessment score, new classification criteria, and performance evaluation based on accuracy, sensitivity, and specificity metric after evaluating studies empirically.

This SLR will help the medical scientists in the targeted identification of cancer, type I diabetic cell for treating the patients, and help the pharmacists in effective drug development by opting the accurate predictor of N-Linked sites. Furthermore, it will facilitate the researchers to develop more accurate and efficient predictive model by analyzing the techniques used by existing researchers.

The proposed article is presented in the following sequence: the methodology adopted to conduct survey along with objectives and research questions is presented in “Survey methodology”. The analysis of the research question is described in “Assessment and discussion”. The “Discussion and future direction” presents synthesis of reviewed literature. Finally, the article has been concluded in “Conclusion”.

## Survey methodology

**Figure 1:**
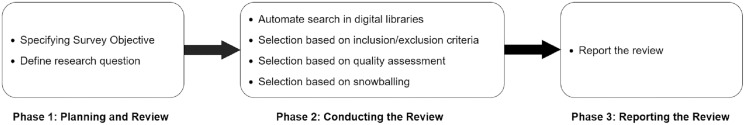
Research strategy.

The survey methodology consists of three phases: plan, conduct of review and conclusion as shown in Fig. 1.

### Review plan

**Figure 2:**
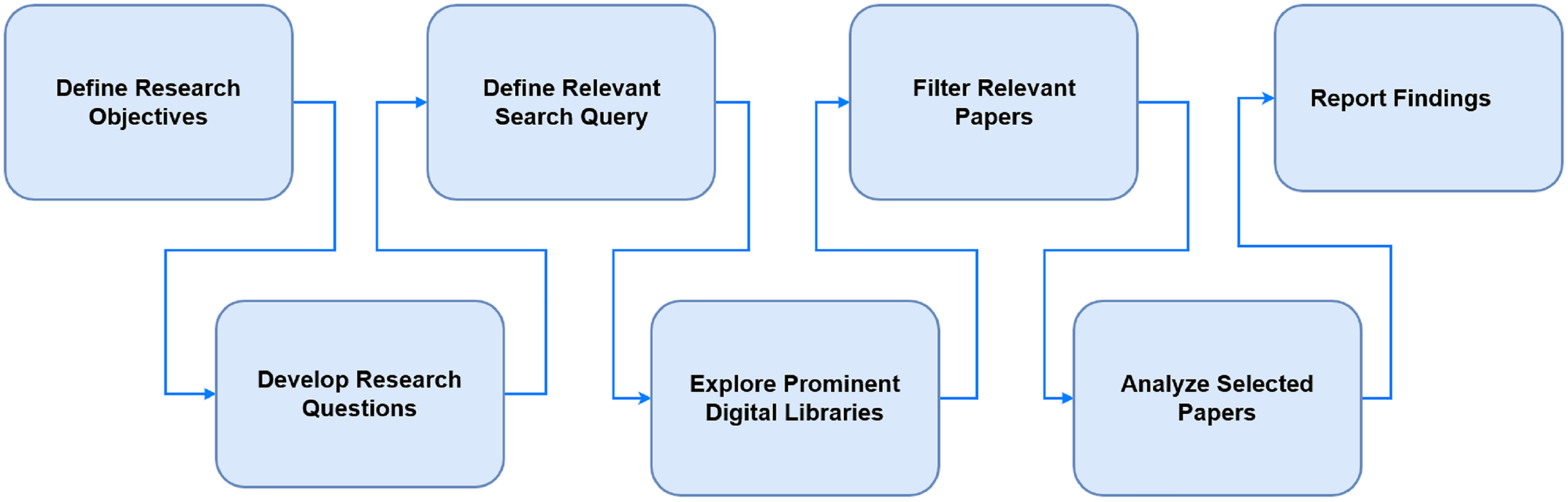
Research strategy.

The process involved to conduct the review is shown in Fig. 2.

### Review conduct

The steps involved to conduct the review were: (a) Search of relevant primary study from different search venues. (b) Selection of relevant research articles from searched articles obtained in previous step through predefined inclusion/exclusion criteria. (c) The selected articles were then assigned score based on their defined quality parameters. (d) Backward snowballing to include the important articles.

#### Automated search in digital library

The relevant research articles have been extracted through system search. Therefore, automatic, and manual search has been performed. The google scholar is used as digital venue to get the relevant research articles.

- Google Scholar (http://scholar.google.com/)
- IEEE Xplore (https://ieeexplore.ieee.org/search/advanced)
- Springer Link (https://link.springer.com/)
- Bioinformatics (https://academic.oup.com/bioinformatics)
- PLOS ONE (https://journals.plos.org/plosone/)

**Figure 3:**
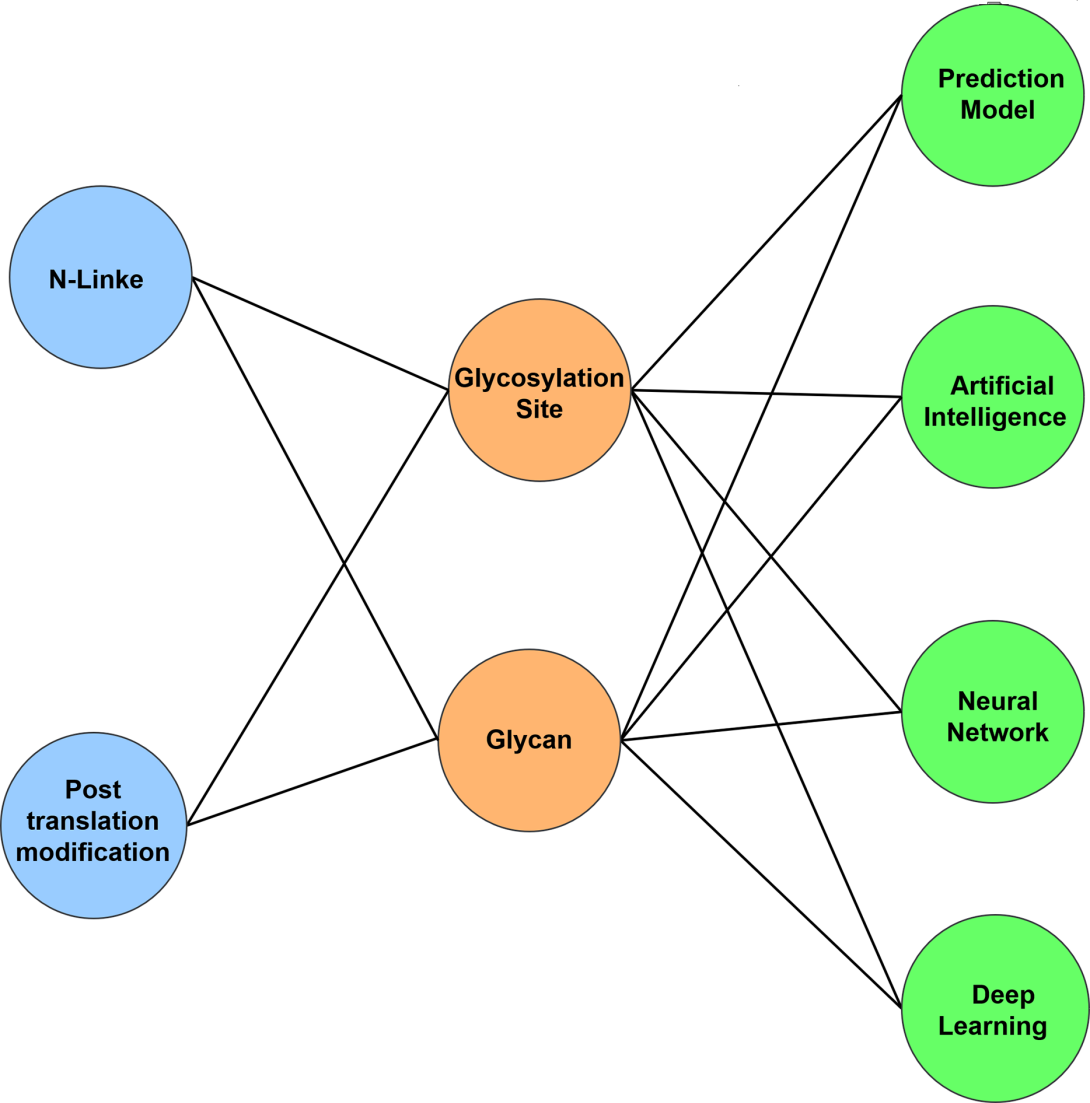
Keyword used to develop query string.

To get appropriate and relevant search result, keyword based search has been applied on the digital venue. Based on the RQs mentioned in Table 2, keyword are selected for primary and secondary term. The Boolean operator ‘AND’ and ‘OR’ are used to build query string. The search query based on keyword is shown in Fig. 3. The search query is grouped into three groups where each group contain the similar keyword to ensure maximum relevant studies as mentioned in Table 1. Using the Boolean operators (OR, AND) final search query is designed in which AND operator is applied in different groups and OR operator is with in different keywords of a group.

**Table 2:** Research questions and objective.

RQ	Research question	Research objective/motivation
RQ1	Which are the relevant publishing channel for N-Linked glycosylation research? Which channel type and geographical area target this research?	To identify • High quality publishing venue. • Research published during 2017–till October-2021. • Scentometric analysis based on meta information including research type, approaches and validation methods.
RQ2	Which are the exiting prediction model (tool) used for the identification of N-linked Glycosylation sites and for which kind of species these sites are identified?	To help the researchers to identify diseases *i.e.*, cancer detection, type 1 diabetic and also drug discoveries through cost effective and time saving approach.
RQ3	Which algorithm or method are used to construct N-Linked feature vector?	To understand the in-depth structure of protein sequences to extract useful information to train model.
RQ4	Which algorithm or method are used to train N-Linked model?	To develop efficient tool to predict the N-linked sites through computational approach.
RQ5	How effective are the existing model to predict the N-Linked sites?	By evaluating the 1. Availability of data set. 2. Availability of tool. 3. Determining the Accuracy measure including Accuracy, Sensitivity and Specificity metrics. 4. Result comparison with existing studies.

Listening 1 [“n linked” OR “Post translation modification”] AND [“Glycosylation sites” OR “Glycan”] AND [“prediction model” OR “Artificial Intelligence” OR “Neural Network” OR “Deep Learning”]

Primary keywords were selected as a key identifier for N-linked prediction models. Primary keywords along with the secondary and additional keywords were chosen. Combination of keywords and Boolean operators have developed as mentioned in Table 3.

**Table 3:** Search group used for search query.

Digital library	Search query	Applied filter
IEEE Xplore	(“n linked” OR “Post translation modification”) AND (“prediction model” OR “Artificial Intelligence” OR “Neural Network” OR “Deep Learning”)	2017–2021
Springer link	(“n linked” OR ”Post translation modification”) AND (“Glycosylation sites” OR “Glycan”) AND (“prediction model” OR “Artificial Intelligence” OR “Neural Network” OR “Deep Learning”)	2017–2021
Bioinformatics	(n linked OR Post translation modification) AND (Glycosylation sites OR Glycan) AND (prediction model OR Artificial Intelligence OR Neural Network OR Deep Learning)	2017–2021
PLOS ONE	(“n linked”) AND (“Glycosylation”) AND (“Neural Network” OR “Deep Learning”)	2017–2021
Google scholar	(“n linked” OR “Post translation modification”) AND (“Glycosylation sites” OR “Glycan”) AND (“prediction model” OR “Artificial Intelligence” OR “Neural Network” OR “Deep Learning”)	2017–2021

#### Inclusion and exclusion criteria for selection

1. Inclusion Criteria
  - The article included in review must contain prediction of N-linked glycosylation sites or Glycosylation sites.
  - It must target any of the research question mentioned in Table 2.
  - It is published in journal or in preprint repository since 2017.
  - It should contain computation or semi computational approach for prediction.

2. Exclusion Criteria
  - Eliminate articles that do not address the N-linked glycosylation or glycosylation.
  - Eliminate articles that purely identify N-linked sites through biological experimentation.
  - Eliminate the books appeared in the result of search query.

#### Quality assessment as selection criteria

The quality assessment (QA) is the major step to conducting any systematic review. In this study, questionnaire has been designed to measure the quality of selected articles. The score is computed on the following criteria:

- The study has awarded score (1) if N-linked predictive tool has developed, otherwise scored (0).
- The study has awarded score (2) if the method developed to extract feature from data based on computational approach, score (1) for hybrid approach and score (0) in-case of experimental approach.
- The study has awarded score (1) if the computation method for training has provided, otherwise scored (0).
- The score (1) has been awarded if the data set used is available otherwise scored (0).
- The score (1) has been awarded if the organism type is available otherwise scored (0).
- The studies were rated by taking conference and journal rating list into account. The possible score for publication is shown in Table 4.

**Table 4:** Possible rating for recognized and stable publication score.

Publication source	+4	+3	+2	+1	0
Journals	Q1	Q2	Q3	Q4	No JCR ranking
Conference	CORE A*	CORE A	CORE B	CORE C	Not in CORE ranking

The resultant score has been calculated for each study by aggregating the points of all question. Article achieving minimum score (5) has been included in the review.

#### Selection based on snowballing

After performing the quality assessment, back-word snowballing to extract the relevant articles from the references of the selected articles. The articles by Kumar et al. (2020) and Ilyas et al. (2019) have been shortlisted after performing the inclusion exclusion criteria and quality assessment.

### Review report

The glycosylation sites especially N-Linked identification is very important domain, therefore in this review, systematic and empirical method is adopted to extract the relevant article from the digital libraries mentioned in Table 3, using query string as shown in Listening 1. Almost 800 articles are left after removing the articles before 2017.

The shortlisted articles are then filtered based on title, abstract, introduction and examined the full article if required for each search result. The article contains less than four pages and irrelevant articles were eliminated. The results of primary search, filtering and inspection phase, covering five digital libraries, are presented in Table 5.

**Table 5:** Selection phase and results.

Phase	Selection	Selection criteria	PLOS ONE	Bioinformatics	Springer link	IEEE Xplore	Google scholar	Total articles
1	Search	Keyword (Fig. 2)	21	3	47	4	770	845
2	Filtering	Title	15	3	18	3	212	251
3	Filtering	Abstract	10	3	13	3	160	189
4	Filtering	Introduction and conclusion	6	2	7	3	125	143
5	Inspection	Full article	1	1	2	2	62	68

After the preprocessing of articles, inclusion/exclusion test has been performed and after that quality assessment score has been computed. The article having at least five score have included in this study and it is total of 70 in count as given in Table 6.

**Table 6:** Classification criteria

Sr. No.	Ref. No.	P.Year	P.Channel	Research type	Empirical type	Species	PTM type	Feature set method	Model training algorithm	Model	(a)	(b)	(c)	(d)	(e)	(f)	SCORE
1	Akmal, Rasool & Khan (2017)	2017	Journal	Solution	Computational	Human	N-linked	Position relative and Statistical Moments	ANN/Back propagation	-	0	2	1	1	1	4	9
2	Chien et al. (2020)	2020	Journal	Solution	Computational	Human and Mouse	N-linked	Sequence, Structure and Function feature	XGBOOST	N-GlycoGo	1	2	1	0	1	4	9
3	Taherzadeh et al. (2019)	2019	Journal	Solution	Computational	Human and Mouse	N-linked and O-linked	Sequence and Structure	Deep ANN and SVM	Sprint-Gly	1	2	1	1	1	4	10
4	Tran, Pham & Ou (2021)	2021	Journal	Solution	Computational	Human and Mouse	N-linked	Word embedding Vector Technique	RM, KNN, SVM and XGBoost.	-	0	2	1	1	0	4	8
5	Liu et al. (2019)	2019	Journal	Solution	Computational	Human	N-linked	Sequence	ANN	NetGlyco (Exiting)	1	2	1	1	1	4	10
6	Li et al. (2019)	2019	Journal	Solution	Computational	Human	N-linked (and C/O-linked)	Sequence and Structure Feature	PA2DE using AlphaMax	GlycoMine_PU	1	2	1	1	1	4	10
7	Bojar et al. (2021b)	2021	Journal	Solution	Hybrid	Eukaryote	Glycosylation	Sequence feature	Recurrent NN (LSTM)	SweetOrigin	0	2	1	1	1	4	9
8	Thomès, Burkholz & Bojar (2021)	2021	Journal	Solution	Computational	Animal	N-linked and O-linked	-	-	GlycoWork	1	2	1	1	1	4	10
9	Carpenter et al. (2022)	2021	bioRxiv	Solution	Computational	Not Mention	Glycosylation	Fingerprint Encoding	MNN (ADAM)	GlyNet	1	2	1	0	1	0	5
10	Pitti et al. (2019)	2019	Journal	Solution	Computational	Human	N-linked	Similarity voiting and Gap Peptide	SVM	NGlyDE	1	2	1	1	1	4	10
11	Lundstrøm et al. (2022)	2021	bioRxiv	Solution	Computational	Human	Glycosylation	Protein-Glycan Sequence Feature	Grpah CNN	LectinOracle	0	2	1	1	1	0	5
12	Burkholz, Quackenbush & Bojar (2021)	2021	Journal	Solution	Computational	Human	Glycosylation	Graph and Statistical feature	Graph NN	SweetNet	1	2	1	1	1	4	10
13	Kotidis & Kontoravdi (2020)	2020	Journal	Solution	Hybrid	Human	N-linked	-	ANN/Kinetic Model	-	0	0	1	1	1	4	7
14	Lee et al. (2021)	2021	Journal	Solution	Experimental	Mammalian	Glycosylation	-	MS	-	0	0	0	0	1	4	5
15	Alkuhlani et al. (2021)	2021	Journal	Review	Computational	Human	Glycosylation	Computational	AI	-	1	2	1		1	4	9
16	Adolf-Bryfogle et al. (2021)	2021	Journal	Solution	Computational	Not Mention	N-linked	KDE	Glycan Tree Modler	Rosetta Carbohydrate Framework	1	2	1	1	0	0	5
17	Sha et al. (2019)	2019	Journal	Solution	Experimental	Human	N-linked	Flux Balance Analysis	Kinetic	-	0	1	0	1	1	2	5
18	Zhang et al. (2021a)	2021	Journal	Solution	Experimental	Human	N-linked	MS	-	-	0	1	0	0	1	4	6
19	Park et al. (2019)	2019	Journal	Solution	Computational	Human	N-linked and O-linked	Sequence and Structure	Clustring	Glycan Reader and Modeler	1	2	1	0	0	4	8
20	Zhang et al. (2021c)	2021	Journal	Solution	Experimental	Human	N-linked	-	-	-	0	0	0	0	1	4	5
21	Xiang, Zou & Zhao (2021)	2021	Journal	Solution	Computational	Human	Glycosylation (O-linked)	feature set selected using SVM them mRmR	SVM,RF and NB	VPTMdb	1	2	1	1	1	4	10
22	Antonakoudis et al. (2021)	2021	Journal	Solution	Hybrid	Human	N-linked	Stochiometric	ANN	-	0	1	1	1	1	4	8
23	Huang et al. (2017)	2017	Journal	Solution	Experimental	Mammalian	N-linked	-	-	-	0	1	0	0	1	4	6
24	Naseer et al. (2021b)	2021	Journal	Solution	Computational	Not Mention	PTM (Amidation)	PseAAC	CNN	IAmideV-deep	1	2	1	1	0	2	7
25	Hwang et al. (2020)	2020	Journal	Solution	Hybrid	Human	N-linked	IQ-GPA human plazma protein	DNN	-	0	1	2	0		4	7
26	Coff et al. (2020)	2020	Journal	Solution	Computational	Human and Avian	Glycosylation	Frequent Subtree mining and mRMR	Regression Classifier	CCARL	1	2	1	1	1	4	10
27	Le, Sandag & Ou (2018)	2018	Journal	Solution	Computational	Human	PTM (including N linked)	Statistical Moment and F score	RBF Network	PTM Transporter	1	2	1	1	1	2	8
28	He, Wei & Zou (2019)	2019	Journal	Review	Computational	Not Mention	N-linked	Provided	Provided	Provided	1	2	1	0	0	4	8
29	Audagnotto & Dal Peraro (2017)	2017	Journal	Review	Computational	Not Mention	N-linked	-	Provided	Provided	1	0	1	1	0	4	7
30	Krasnova & Wong (2019)	2019	Journal	Review	Experimental	Human	N-linked and O-linked	-	-	-	0			0	1	4	5
31	Kellman & Lewis (2021)	2020	Journal	Solution	Experimental	Human	Glycan (including N)	-	-	-	1			1		4	6
32	Huang et al. (2021)	2021	Journal	solution	Computational	Not Mention	Glycosylation (O-linked)	Sequence feature	RF	OGP-Based	1	2	1	1	0	4	9
33	Shek, Kotidis & Betenbaugh (2021)	2021	Journal	Review	Computational	Not Mention	Glycosylation	-	Provided	Provided	1	2	1	0	0	4	8
34	Mondragon-Shem et al. (2020)	2020	Journal	solution	Hybrid	Human	N-linked	MS	-	Existing Tool	1	1	0	1	1	4	8
35	Wilson et al. (2021)	2021	Journal	solution	Experimental	Human	Glycosylation (including N)	-	-	-	0	1	0	1	1	2	5
36	Zhang et al. (2021b)	2021	Journal	solution	Computational	Mammalian	N-linked	Unknown Parameter and Structure	Baysen Network	-	0	1	1	0	1	4	7
37	Hua11 (2019)	2019	Conference	Solution	Computational	Human	Protein Prediction	Frequency Feature of AA and EH Method	SVM and NN	PPSNN	1	2	1	0	1	0	5
38	Zhao et al. (2020)	2020	Journal	Solution	Experimental	Human	N-linked	-	MS	-	0	0	1	0	1	4	6
39	Wang et al. (2017)	2017	Journal	solution	Hybrid	human	N-linked	CfsSubSetEval	SVM	-	0	1	2	0	1	4	8
40	Badgett et al. (2018)	2018	Journal	Solution	Experimental	Human	N-linked	-	MS	-	0	0	1	1	1	2	5
41	Suga, Nagae & Yamaguchi (2018)	2018	Journal	Solution	Hybrid	Human	N-linked	Structural Feature	Maturation	-	0	1	1	1	1	4	8
42	Bao et al. (2019)	2019	Journal	Solution	Computational	Human	Glycosylation and Phosphorylation	Membrane Buried, Confrontational and average Flexible Indices	NN+ELM+SVM	CMSENN	1	2	1	0	1	3	8
43	de Souza et al. (2019)	2019	Journal	Solution	Experimental	human	N-linked	-	MS	-	0	0	0	1	1	4	6
44	Jiang et al. (2018)	2018	Journal	Solution	Computational	Not Mention	Glycosylation (O-linked)	KPCA and FUS	Rotation Forest	OGLYCPred	1	2	1	1		4	9
45	Kuo-Chen (2019)	2019	Journal	Review	Computational	Human	Non-Glycosylation	KC Chou’s 5 step	-	Povided	1	1	0	1	1	1	5
46	Dimeglio et al. (2020)	2020	Journal	Solution	Hybrid	Human	N-linked	Statistical Moment	ANN	THETA Model	1	2	1	1	1	4	10
47	Dobson, Zeke & Tusnády (2021)	2021	Journal	Solution	Hybrid	Human	Protein Traffic membrane (N and O)	Topology and Putative SLiMs	CNN with Adam	PolarportPred	1	1	1	0	1	4	8
48	Kumar et al. (2020)	2020	Conference	Solution	Hybrid	Human	PTM	Psycho-Chemical, structural and PTM	ML	-	0	2	1	1	1	0	5
49	Ilyas et al. (2019)	2019	Journal	solution	Computational	Human	PTM	Chou’s 5-steps	ANN	-	0	2	1	1	1	2	7
50	Yang & Han (2017)	2017	Journal	Solution	Computational	Mammalian	Glycosylation (O-linked)	Protein factor base Features	KNN	-	0	2	1	0	1	2	6
51	Magaret et al. (2019)	2019	Journal	Solution	Hybrid	Not Mention	N-linked	Sequences	RM, Super Learner and Glmnet	-	0	1	1	1	0	4	7
52	Sugár et al. (2021)	2021	Journal	Solution	Experimental	Human	N-linked	-	RanoLC-MS	-	1	1	0	1	1	4	8
53	Campbell (2017)	2017	Journal	review	Hybrid	Human	Glycosylation	Partial Mentioned	Partial Mentioned	-	1	1	1	0	1	1	5
54	Jia, Zuo & Zou (2018)	2018	Journal	Solution	Computational	Not Mention	Glycosylation (O-linked)	FUS and KPCA	KNN,RM,SVM and NB, SVM outperform	rgb 0.141, 0.125, 0.129O-GlcNAcPRED-II	1	2	1	1	0	4	9
55	Ferreira et al. (2021)	2021	Journal	Solution	Experimental	Human	N-linked	-	MS	-	0	1	0	0	1	4	6
56	Ye & Vakhrushev (2021)	2021	Journal	Solution	Experimental	Human	Glycosylation	-	MS	-	0	0	0	0	1	4	5
57	Bojar et al. (2021a)	2021	bioRxiv	Solution	Hybrid	Human	N-linked	Sequence	ML	-	0	2	1	1	1	0	5
58	Desaire, Patabandige & Hua (2021)	2021	Journal	Solution	Hybrid	Not Mention	Glycosylation	MS	SVM	-	0	1	1	1	0	4	7
59	Chen et al. (2021)	2021	Journal	Solution	Computational	Human	PTM	Binary Encoding,AAC,EAAC and Dipeptide	Deep Learning	CNNrgb	1	2	1	1	1	4	10
60	Zou et al. (2017)	2017	Conference	Solution	Computational	Human	Glycosylation (O-linked)	Vector Word	SVM	GLycoCell	1	2	1	1	1	0	6
61	Perpetuo et al. (2021)	2021	Journal	Solution	Computational	Human	PTM	Sequences	AI	-	0	2	1	0	1	3	7
62	Li et al. (2020)	2020	Journal	Solution	Computational	Not Mention	Protein	AAC, PseAAC,NC, PseKNC	adaboost and random forest	PPAI	1	2	1	0	0	4	8
63	Lei, Tang & Du (2017)	2017	Journal	Solution	Hybrid	Not Mention	PTM (S-sulfenylated)	Psysciochemical and Clustring Method	Ensemble Classifier	-	0	1	2	1	0	1	5
64	Murad et al. (2021)	2021	bioRxiv	Solution	Computational	Not Mention	PTM (Ubiquitination)	Statistical Moment	Random Forest	UBISites-SRF	1	2	1	0	1	0	5
65	Qiu et al. (2018)	2018	Journal	Solution	Computational	Not Mention	PTM (Lipoylation)	Biprofile Bayes Encoding	SVM	LipoPred	1	1	1	0	0	3	6
66	Yang et al. (2019)	2019	Journal	Solution	Hybrid	Human	PTM	SNP	-	Awesome	1	1	1	1	1	4	9
67	Liu et al. (2021)	2021	Journal	Solution	Computational	Human	PTM	UbiSite-XGBoost	Extreme gradient boosting classifier	UbiSite=XGBoost	1	2	1	1	1	3	9
68	Ruiz-Blanco et al. (2017)	2017	Journal	Solution	Computational	Human	N-linked	ProDCal	Jrip Classifier	Sequon	0	2	1	1	1	4	9
69	Kumari, Kumar & Kumar (2018)	2018	Journal	Solution	Computational	Human	Palmitoylation	PSSM	SVM	RAREPalm	1	2	1	0	1	3	8
70	Huang & Li (2018)	2018	Journal	Solution	Computational	Human	PTM	Sequence, Structure	KNN	-	0	2	1	1	1	4	9

## Assessment and discussion

In this section, the research questions have been analyzed based on 70 primary studies.

## Assessment of q1:

### Which are the relevant publishing channel for N-linked glycosylation research? Which channel type and geographical area target this research?

To find the relevant publishing channel, channel type and geographical aspects for the N-linked glycosylation sites requires the meta information. To achieve this purpose, channel type, publishing year and demographical distribution is presented for the analysis of selected studies.

**Figure 4:**
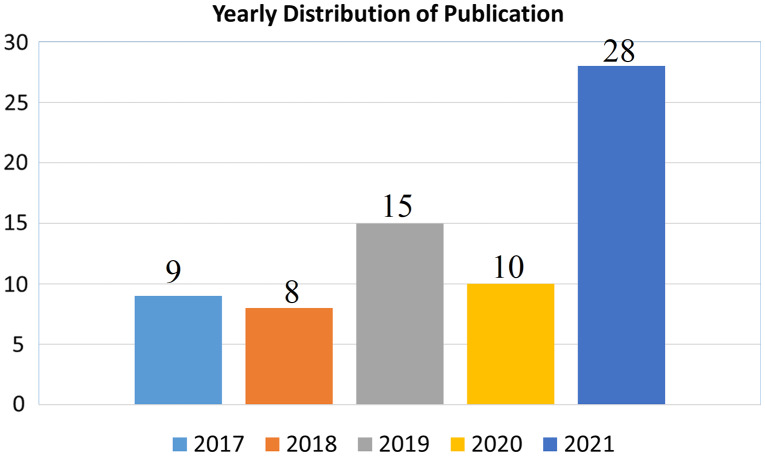
Year wise distribution of publication.

The importance of selected topic can be evaluated from the yearly publication on the relevant domain. The 28 out of 70 articles has been published in 2021 which also of 40% of selected article as shown in Fig. 4.

**Figure 5:**
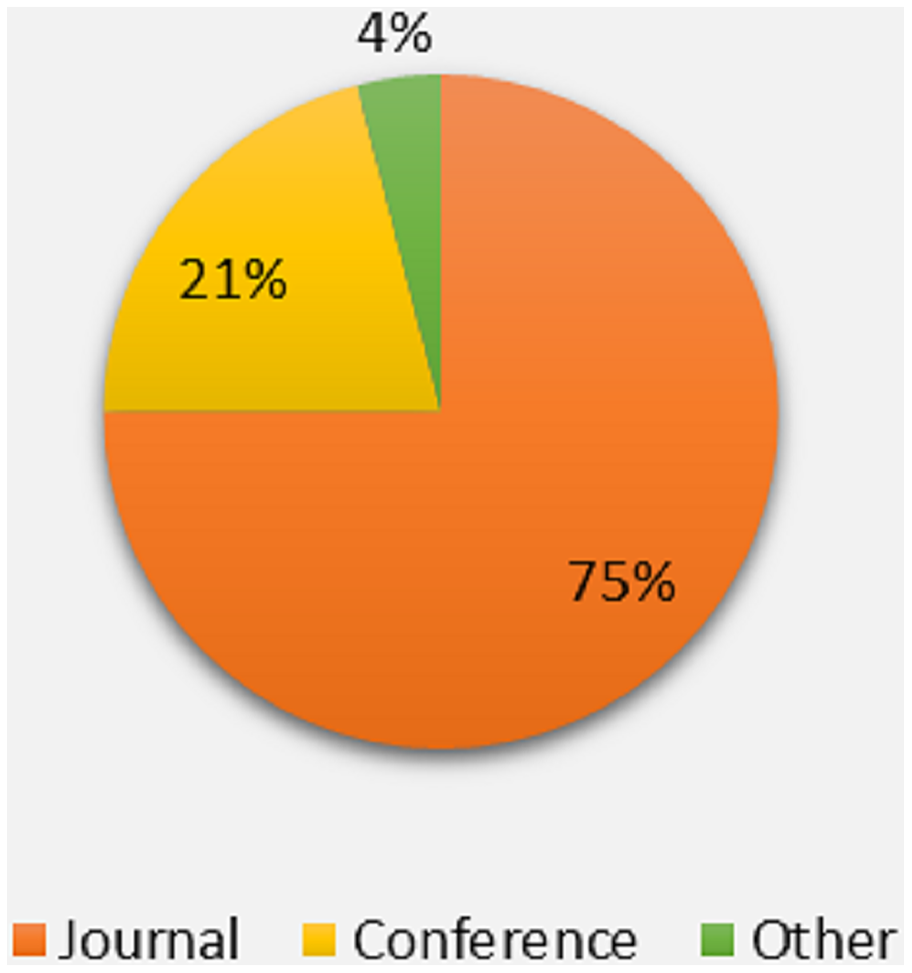
Percentage of publication channel.

It is clear from Fig. 5 that the maximum portion of studies belong to the recognized journal followed by international conferences.

**Figure 6:**
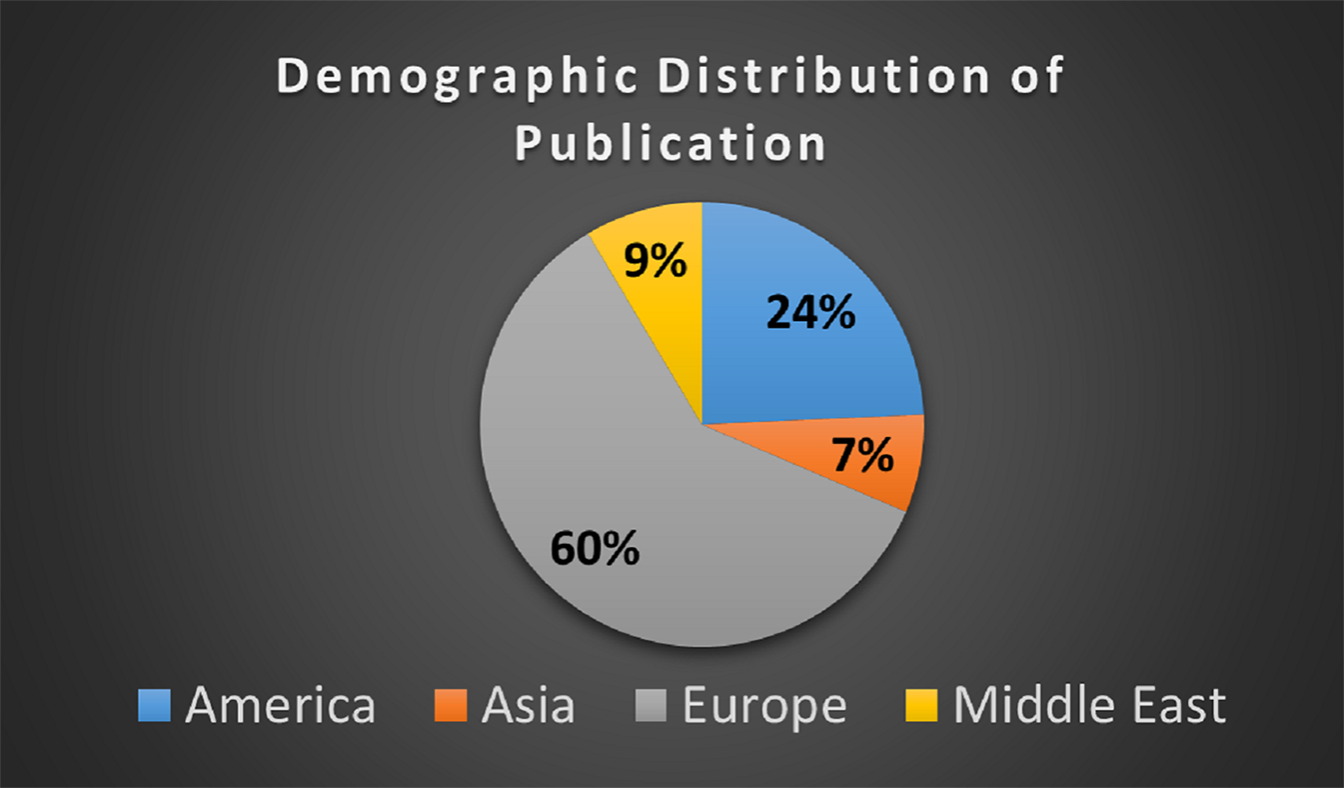
Demographical distribution of publication.

It is observed, 42 out of 70 studies have been published in the different regions of the Europe as shown in Fig. 6.

Quality assessment score for each finalized study awarded according to defined criteria in quality assessment score section, shown in Table 7. It is clearly observed that only studies qualifying minimum threshold are listed. The article published in Q1 quality journal achieve highest score, it will help researchers to find the relevant publishing venues for the N-linked and other glycosylation site prediction studies. Almost 50% of the studies achieve eight score or above which shows the relevancy of the selected studies through developed query string.

**Table 7:** Quality assessment score.

Reference	QA Score	Total articles
Taherzadeh et al. (2019), Liu et al. (2019), Li et al. (2019), Thomès, Burkholz & Bojar (2021), Pitti et al. (2019), Burkholz, Quackenbush & Bojar (2021), Xiang, Zou & Zhao (2021), Coff et al. (2020), Dimeglio et al. (2020), Chen et al. (2021)	10	10
Akmal, Rasool & Khan (2017), Chien et al. (2020), Bojar et al. (2021b), Alkuhlani et al. (2021), Huang et al. (2021), Jiang et al. (2018), Jia, Zuo & Zou (2018), Yang et al. (2019), Liu et al. (2021), Ruiz-Blanco et al. (2017), Huang & Li (2018)	9	11
Tran, Pham & Ou (2021), Park et al. (2019), Antonakoudis et al. (2021), Le, Sandag & Ou (2018), He, Wei & Zou (2019), Shek, Kotidis & Betenbaugh (2021), Mondragon-Shem et al. (2020), Wang et al. (2017), Suga, Nagae & Yamaguchi (2018), Bao et al. (2019), Dobson, Zeke & Tusnády (2021), Sugár et al. (2021), Li et al. (2020), Kumari, Kumar & Kumar (2018)	8	14
Kotidis & Kontoravdi (2020), Naseer et al. (2021b), Hwang et al. (2020), Audagnotto & Dal Peraro (2017), Zhang et al. (2021b), Ilyas et al. (2019), Magaret et al. (2019), Desaire, Patabandige & Hua (2021), Perpetuo et al. (2021)	7	9
Zhang et al. (2021a), Huang et al. (2017), Kellman & Lewis (2021), Zhao et al. (2020), de Souza et al. (2019), Yang & Han (2017), Ferreira et al. (2021), Zou et al. (2017), Qiu et al. (2018)	6	9
Carpenter et al. (2022), Lundstrøm et al. (2022), Lee et al. (2021), Adolf-Bryfogle et al. (2021), Sha et al. (2019), Zhang et al. (2021c), Krasnova & Wong (2019), Wilson et al. (2021), Hua11 (2019), Badgett et al. (2018), Kuo-Chen (2019), Kumar et al. (2020), Campbell (2017), Ye & Vakhrushev (2021), Bojar et al. (2021a), Lei, Tang & Du (2017), Murad et al. (2021)	5	17

The overall classification result and QA studies have presented in of Table 6. The finalized articles have classified based on seven parameters: research type (solution proposed or review article), empirical type (computational approach, experimental approach based on biological studies or hybrid approach based on computational and biological study), glycosylation type, specie type, method (used for feature extraction), Algorithm (used to train predictive model) and tool (developed for prediction).

Furthermore, the sources of finalized studies, and total number/percentage of studies per publication source mentioned in Table 8.

**Table 8:** Percentage count of articles published in channel.

Publication source	Reference	Count	%age
Amino Acids	Ruiz-Blanco et al. (2017)	1	1
Analytical and Bioanalytical Chemistry	Desaire, Patabandige & Hua (2021)	1	1
Bioinformatics	Taherzadeh et al. (2019), Jiang et al. (2018), Dimeglio et al. (2020), Dobson, Zeke & Tusnády (2021), Jia, Zuo & Zou (2018)	5	7
bioRxiv	Carpenter et al. (2022), Lundstrøm et al. (2022), Adolf-Bryfogle et al. (2021), Bojar et al. (2021a), Murad et al. (2021)	5	7
Biotechnology and Bioengineering	Zhang et al. (2021b)	1	1
BMC Bioinformatics	Li et al. (2019), Coff et al. (2020), Li et al. (2020)	3	4
Briefings in Bioinformatics	Xiang, Zou & Zhao (2021)	2	3
Briefings in Functional Genomics	He, Wei & Zou (2019)	1	1
Cell Host Microbe	Bojar et al. (2021b)	1	1
Cell Reports	Burkholz, Quackenbush & Bojar (2021)	1	1
Chemometrics and Intelligent Laboratory Systems	Bao et al. (2019), Qiu et al. (2018)	2	3
Computational and Structural Biotechnology Journal	Audagnotto & Dal Peraro (2017)	1	1
Computational Biology and Chemistry	Le, Sandag & Ou (2018), Yang & Han (2017)	2	3
Computers Chemical Engineering	Antonakoudis et al. (2021)	1	1
Computers in Biology and Medicine	Tran, Pham & Ou (2021)	1	1
Current Bioinformatics	Alkuhlani et al. (2021), Huang & Li (2018)	2	3
Current Genomics	Ilyas et al. (2019)	1	1
Current Opinion in Chemical Engineering	Shek, Kotidis & Betenbaugh (2021)	1	1
Environmental Microbiology	Zhang et al. (2021c)	1	1
Expert Review of Proteomics	Perpetuo et al. (2021)	1	1
Frontiers in Endocrinology	Zhang et al. (2021c)	1	1
Fuzzy Systems and Data Mining	Hua11 (2019)	1	1
Genomics, Proteomics Bioinformatics	Huang et al. (2021), Ferreira et al. (2021)	2	3
Glycobiology	Thomès, Burkholz & Bojar (2021), Park et al. (2019), Suga, Nagae & Yamaguchi (2018)	3	4
IEEE Access	Chien et al. (2020)	1	1
IEEE International Conference on Machine Learning and Applied Network Technologies (ICMLANT)	Kumar et al. (2020)	1	1
International Conference of Pioneering Computer Scientists, Engineers and Educators. Springer, Singapore,	Zou et al. (2017)	1	1
Journal of Bimolecular Techniques	Badgett et al. (2018)	1	1
Journal of Computational Biology	Kumari, Kumar & Kumar (2018)	1	1
Journal of Molecular Graphics and Modelling	Liu et al. (2021)	1	1
Journal of Proteomics	Zhao et al. (2020), de Souza et al. (2019)	2	3
Journal of the American Chemical Society	Huang et al. (2017), Krasnova & Wong (2019)	2	3
Letters in Organic Chemistry	Lei, Tang & Du (2017)	1	1
Mathematical Bioscience	Liu et al. (2019)	1	1
Metabolic Engineering Communications	Kotidis & Kontoravdi (2020)	1	1
Molecular Cellular Proteomic	Ye & Vakhrushev (2021)	1	1
Nature Communications	Wang et al. (2017)	1	1
Nucleic Acids Research	Yang et al. (2019)	1	1
PLoS Computational Biology	Magaret et al. (2019)	1	1
PLOS ONE	Akmal, Rasool & Khan (2017)	1	1
Processes	Sha et al. (2019)	1	1
Scientific Reports	Pitti et al. (2019), Hwang et al. (2020), Mondragon-Shem et al. (2020), Sugár et al. (2021)	4	6
Symmetry	Naseer et al. (2021b)	1	1
The American Journal of Human Genetics	Wilson et al. (2021)	1	1
Trends Artifi. Intell	Kuo-Chen (2019)	1	1
Trends in Biochemical Science	Kellman & Lewis (2021)	1	1
Trends in Glycoscience and Glycotechnolog	Campbell (2017)	1	1
Trends in Microbiology	Lee et al. (2021)	1	1

## Assessment of q2:

### Which are the exiting prediction model (tool) for the identification of N-linked Glycosylation sites and for which kind of species these sites are identified?

The available tool to identify the N-Linked glycosylation sites and for which kind species it can identify the relevant site is the parameter of this study. There is hierarchy of N-Linked Glycan to PTM. Where PTM is classified into various type and Glycosylation in one of them and glycosylation is further classified into five group and N-linked is one of them.

**Figure 7:**
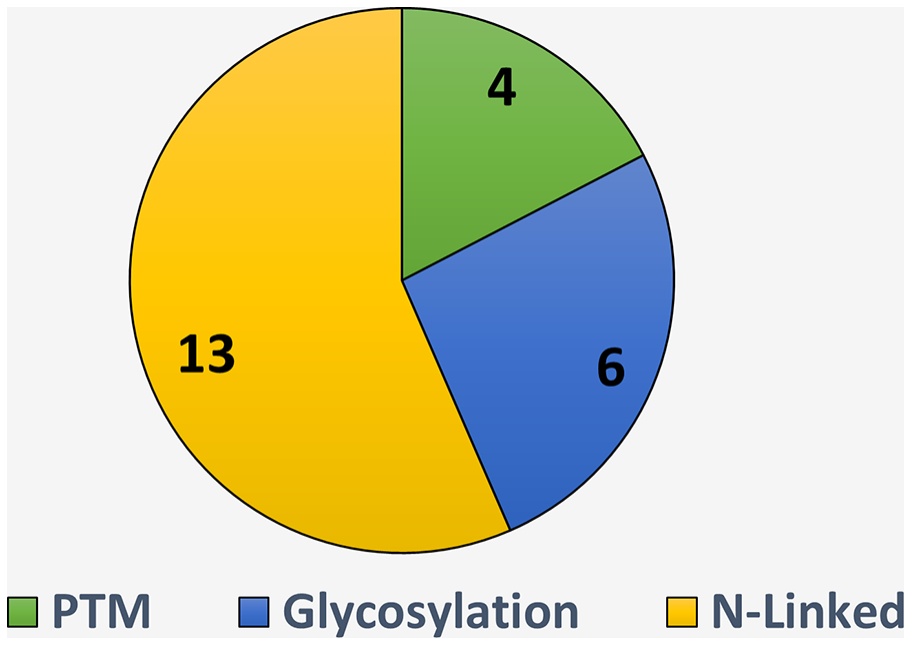
Tool available for N-linked sites identification.

The summarized detail of eight is represented in Fig. 7. It is observed, there are 13 studies including (Chien et al., 2020; Taherzadeh et al., 2019; Liu et al., 2019; Li et al., 2019; Thomès, Burkholz & Bojar, 2021; Pitti et al., 2019; Adolf-Bryfogle et al., 2021; Park et al., 2019; He, Wei & Zou, 2019; Audagnotto & Dal Peraro, 2017; Mondragon-Shem et al., 2020; Dimeglio et al., 2020; Ruiz-Blanco et al., 2017) which have developed the tool specific to the N-Linked site identifications, few studied developed tool for glycosylation sites identification irrespective of the specific type including (Bojar et al., 2021b; Carpenter et al., 2022; Lundstrøm et al., 2022; Burkholz, Quackenbush & Bojar, 2021; Coff et al., 2020; Shek, Kotidis & Betenbaugh, 2021) and some authors (Le, Sandag & Ou, 2018; Liu et al., 2021; Yang et al., 2019; Campbell, 2017) develop tool without mentioning the type of PTM. These all tools have list down in the Table 9.

**Table 9:** N-Linked glycosylation available tool.

Ref.	P.Year	Species	Tool	Finding
Bojar et al. (2021b)	2021	Eukaryote	SweetOrigin	The model develop to identify Glycosylation sites using Hybrid approach on Eukaryotes.
Thomès, Burkholz & Bojar (2021)	2021	Animal	GlycoWork	The computational model used to identify both N and O-linked in Animal.
Carpenter et al. (2022)	2021	Not mention	GlyNet	The computational model used to identify glycosylation protein sequences.
Lundstrøm et al. (2022)	2021	Human	LectinOracle	The computational model used to identify glycosylation protein sequences for human.
Burkholz, Quackenbush & Bojar (2021)	2021	Human	SweetNet	The computational model used to identify glycosylation protein sequences for human.
Adolf-Bryfogle et al. (2021)	2021	Not mention	Rosetta Carbohydrate Framework	The computational model used to identify N-linked sites and species are not mentioned.
Shek, Kotidis & Betenbaugh (2021)	2021	Not mention	Provided	The computational model used to identify glycosylation sites and species are not mentioned.
Chen et al. (2021)	2021	Human	CNNrgb	The computational model used to identify PTM sites for human protein.
Liu et al. (2021)	2021	Human	UbiSite = XGBoost	The computational model used to identify PTM sites for human protein.
Chien et al. (2020)	2020	Human and Mouse	N-GlycoGo	The computational model used to identify N-Linked sites for human and mouse protein sequences.
Coff et al. (2020)	2020	Human and avian	CCARL	The computational model used to identify glycosylation sites for human and avian protein sequences.
Mondragon-Shem et al. (2020)	2020	Human	Existing Tool	The hybrid model consists of both experimental and computational approach to develop N-linked site identification on human protein
Dimeglio et al. (2020)	2020	Human	THETA Model	The hybrid model consists of both experimental and computational approach to develop N-linked site identification on human protein
Taherzadeh et al. (2019)	2019	Human and Mouse	Sprint-Gly	The computational model used to identify both N and O-linked in human and Mouse.
Liu et al. (2019)	2019	Human	NetGlyco (Exiting)	The computational model used to identify N-linked sites in human.
Li et al. (2019)	2019	Human	GlycoMine_PU	The computational model used to identify N, O and C-linked in human.
Pitti et al. (2019)	2019	Human	NGlyDE	The computational model used to identify N-linked in human.
Park et al. (2019)	2019	Human	Glycan Reader and Modeler	The computational model used to identify both N and O-linked in human.
He, Wei & Zou (2019)	2019	Not mention	Provided	The computational model used to identify N-linked sites while specie is not mentioned.
Yang et al. (2019)	2019	Human	Awesome	The hybrid approach develop to identify PTM sites for human.
Le, Sandag & Ou (2018)	2018	Human	PTM Transporter	The computational approach developed PTM sites including N-Linked sites for human.
Audagnotto & Dal Peraro (2017)	2017	Not mention	Provided	The computational model used to identify N-linked sites while specie type is missing.
Ruiz-Blanco et al. (2017)	2017	Human	Sequon	Computational method to identify N-Linked sites for human.

It is important to specify for which kind of species these tools will be operating, therefore to achieve this purpose the information is also extracted from the selected studies. Some authors (He, Wei & Zou, 2019; Audagnotto & Dal Peraro, 2017; Shek, Kotidis & Betenbaugh, 2021; Carpenter et al., 2022) did not mention the organism type while other mentioned it and it is observed most of them use human data for site identification as mention in Table 9.

## Assessment of q3:

### Which algorithm or method are used to construct N-Linked feature vector?

The data is the major component to develop any machine learning model (Mahmood et al., 2020; Naseer et al., 2020a, 2020b; Khan et al., 2020b). In bioinformatics, there are two major sources of data on which model can be developed, one is existing repositories such as UniProt (protein repository), GenBank (nucleotide sequence) etc. and other is experimental data which obtain from specific biological experiments. The dataset obtained from any source needs preprocessing to construct the feature vector. The more accurate feature helps to develop efficient model (Barukab et al., 2019; Butt & Khan, 2019; Hussain, Rasool & Khan, 2020; Shah & Khan, 2020). For this purpose, feature method used to predict the N-Linked sites in the selected articles have taken as a parameter of this study.

Most of the authors used the computational feature extraction approach while few used the experimental data obtained from mass spectrometry, human plasma and psycho-chemical method as mentioned in Table 10. It is observed, mostly researcher (Akmal, Rasool & Khan, 2017; Chien et al., 2020; Taherzadeh et al., 2019; Liu et al., 2019; Li et al., 2019; Bojar et al., 2021b; Lundstrøm et al., 2022; Park et al., 2019; Le, Sandag & Ou, 2018; Suga, Nagae & Yamaguchi, 2018; Dimeglio et al., 2020; Magaret et al., 2019; Kumar & Gilula, 1986; Perpetuo et al., 2021; Huang & Li, 2018) used the statistical moment method based on combination of protein sequence, structure and functions along with some other parameters like position relevance of sequences using the protein dataset to construct the feature matrix. The other computational method used to construct features selected article are word embedding vector technique, UbiSite-XGBoost, Similarity voting, CfsSubSetEval, Kernel Density Estimate, correlation subset and graph method as mentioned in Table 10.

**Table 10:** Feature methods for the N-linked sites identification.

Ref.	Glyotype	Method for feature	Finding
Tran, Pham & Ou (2021)	N-Linked	Word embedding Vector Technique	Word embedding technique to efficiently predict N-linked glycosylation sites in ion channels.
Adolf-Bryfogle et al. (2021)	N-Linked	KDE	Kernel Density Estimation based feature extracted.
Antonakoudis et al. (2021)	N-Linked	Stoichiometric	Hybrid method that used the experimental data using stoichiometric.
Zhang et al. (2021b)	N-Linked	Unknown Parameter and Structure	Protein structure feature and some undefined features used to construct feature vector.
Bojar et al. (2021a)	N-Linked	Sequence	Sequence based features computed.
Chien et al. (2020)	N-Linked	Sequence, Structure and Function feature	sequence, structure and function base feature set of human and mouse used to predict site on imbalance dataset.
Hwang et al. (2020)	N-Linked	IQ-GPA human plasma protein	IQ-GPA procedure was used to obtain data from human plasma.
Mondragon-Shem et al. (2020)	N-Linked	MS	Hybrid method based on Mass Spectrometry used data used for training.
Liu et al. (2019)	N-Linked	Sequence	Sequence based protein sequences have computed.
Pitti et al. (2019)	N-Linked	Similarity voting and Gap Peptide	Similarity Voting method and gap peptide method used to construct features.
Magaret et al. (2019)	N-Linked	Sequences	Sequence based protein sequences have computed.
Suga, Nagae & Yamaguchi (2018)	N-Linked	Structural Feature	Structure based protein sequences have computed.
Akmal, Rasool & Khan (2017)	N-Linked	Position relative and Statistical Moments	Position relative features and statistical moment based features have computed.
Wang et al. (2017)	N-Linked	CfsSubSetEval	Patients with different drug responses
Ruiz-Blanco et al. (2017)	N-Linked	ProDCal	ProtDCal method used to get protein features.
Dimeglio et al. (2020)	N-Linked	Statistical Moment	Statistical Moments computed to construct feature vector.
Li et al. (2019)	N-Linked (and C/O-Linked)	Sequence and Structure Feature	Sequence and structure based protein sequences have computed.
Taherzadeh et al. (2019)	N-Linked and O-Linked	Sequence and Structure	Sequence and structure based protein sequences have computed.
Park et al. (2019)	N-Linked and O-Linked	Sequence and Structure	Sequence and structure based protein sequences have computed.
Bojar et al. (2021b)	Glycosylation	Sequence feature	Develop models for glycans that are trained on a curated dataset of 19,299 unique glycans and used sequence based features.
Carpenter et al. (2022)	Glycosylation	Fingerprint Encoding	Feature vector based on Fingerprint encoding method for Predicting Protein-Glycan Interaction
Lundstrøm et al. (2022)	Glycosylation	Protein-Glycan Sequence Feature	The sequence feature of combined protein and glycan are used to extract feature vector based on sequence features.
Burkholz, Quackenbush & Bojar (2021)	Glycosylation	Graph and Statistical feature	Graph algorithm and statistical moments are used to construct feature matrix for glycan.
Desaire, Patabandige & Hua (2021)	Glycosylation	MS	Hybrid method based on Mass Spectrometry used data used for training.
Coff et al. (2020)	Glycosylation	Frequent Subtree mining and mRMR	frequent subtree mining and mRMR used for feature vector construction.
Perpetuo et al. (2021)	PTM	Sequences	Sequence based features used for feature vector construction.
Liu et al. (2021)	PTM	UbiSite-XGBoost	Pseudo ACC, K-spaced Acid Pair, Adapted Normal Distribution bi-profile Bayes, AA Index, Encoding Based Group Weight, LASSO, SMOTE and eXtreme Gradient Boosting features methods are used.
Kumar et al. (2020)	PTM	Psycho-Chemical, structural and PTM	Psycho-Chemical, structure moment of protein and PTM sequence features were used.
Ilyas et al. (2019)	PTM	Chou’s 5-steps	Chou’s 5-steps based feature vector was used.
Yang et al. (2019)	PTM	SNP	Single Nucleotide Polymorphism approach used to compute features.
Huang & Li (2018)	PTM	Sequence, Structure	Sequence and Structure based protein sequences have computed.
Chen et al. (2021)	PTM	Binary Encoding, AAC, EAAC and Dipeptide	Various features have extracted including binary encoding, Amino Acid Composition, Enhanced AAC and Dipeptide.
Le, Sandag & Ou (2018)	PTM (including N Linked)	Statistical Moment and F score	Statistical moment used and then F-Score was computed

## Assessment of q4:

### Which algorithm or method are used to train N-Linked computation model?

The choice of algorithm to train any predictive model is most important factor which impact the performance of any model (Butt & Khan, 2019; Hussain, Rasool & Khan, 2020; Malebary & Khan, 2021). Therefore, it is required to know which type of algorithm are being used to develop the N-linked prediction model. For this purpose, algorithm used for training models in the selected article has been noted as the parameter of this review article as mentioned in Table 11.

**Table 11:** Training algorithm (method) used for N-linked model.

Ref.	Model training algorithm	PTM type	Finding
Akmal, Rasool & Khan (2017)	ANN/Back propagation	N-Linked	Prediction of N-linked glycosylation sites using position relative features and statistical moments through multilayered ANN using back propagation approach.
Chien et al. (2020)	XGBOOST	N-Linked	Extreme Gradient Boost method was used to predict site on imbalance dataset.
Tran, Pham & Ou (2021)	RF, KNN, SVM and XGBoost	N-Linked	Various classifiers were used for prediction including Random Forest, K-Nearest Neighbor, Support Vector Machine and XGBoost but RM outperform.
Liu et al. (2019)	ANN	N-Linked	Artificial Neural Network algorithm used to identify N-linked site in Influenza virus using existing model on dataset.
Pitti et al. (2019)	SVM	N-Linked	N-GlyDE: a two-stage N-linked glycosylation site prediction incorporating gapped dipeptides and pattern-based encoding using SVM after collecting feature vector through two stages.
Kotidis & Kontoravdi (2020)	ANN/Kinetic Model	N-Linked	artificial neural networks and Kinetic model used for predicting protein glycosylation.
Adolf-Bryfogle et al. (2021)	Glycan Tree Modler	N-Linked	prediction based on Tree method.
Sha et al. (2019)	Kinetic	N-Linked	a two-component modeling framework integrating FBA and glycosylation kinetic model was used for prediction.
Antonakoudis et al. (2021)	ANN	N-Linked	predict N linked sites using features computed by stoichiometric and then train model using ANN with forward propagation.
Hwang et al. (2020)	DNN	N-Linked	N linked site using DNN which later used to classify fucosylation
Zhang et al. (2021b)	Baysen Network	N-Linked	Probabilistic model by Bayesian network for the prediction of antibody glycosylation in perfusion and fed-batch cell cultures
Wang et al. (2017)	SVM	N-Linked	Drug responses identified using SVM method.
Dimeglio et al. (2020)	ANN	N-Linked	New genotypic approach for predicting HIV-1 CRF02-AG using ANN
Magaret et al. (2019)	RM, Super Learner and Glmnet	N-Linked	Protein sequence and biological data used to identify N-linked sites using super learner algorithm.
Bojar et al. (2021a)	ML	N-Linked	Guide to Lectin Binding: Machine-Learning Directed Annotation of 57 Unique Lectin Specificities
Ruiz-Blanco et al. (2017)	Jrip Classifier	N-Linked	Novel “extended sequons” of human N-glycosylation sites improve the precision of qualitative predictions: an alignment-free study of pattern recognition using ProtDCal protein features.
Park et al. (2019)	Clustering	N-O Linked	CHARMM-GUI Glycan Modeler for modeling and simulation of carbohydrates and glycoconjugates.
Dobson, Zeke & Tusnády (2021)	CNN with Adam	N-O Linked	Novel mechanism to collect dataset using polarization and then train on CNN model.
Taherzadeh et al. (2019)	Deep ANN and SVM	N-O Linked	Predicting N-and O-linked glycosylation sites of human and mouse proteins by using sequence and predicted structural properties through DNN and SVM
Li et al. (2019)	PA2DE using AlphaMax	N-C-O Linked	Positive-unlabeled data set used to predict sites using AlphaMax algorithm
Bojar et al. (2021b)	Recurrent NN (LSTM)	Glycosylation	develop deep-learning using Recurrent NN models used for glycans that are trained on a curated dataset of 19,299 unique glycans and can be used to study and predict glycan functions.
Carpenter et al. (2022)	MNN (ADAM)	Glycosylation	A Multi-Task Neural Network using ADAM algorithm used for Predicting Protein-Glycan Interaction
Lundstrøm et al. (2022)	Graph CNN	Glycosylation	LectinOracle, a model combining transformer-based representations for proteins and graph convolutional neural networks for glycans to predict their interaction.
Burkholz, Quackenbush & Bojar (2021)	Graph NN	Glycosylation	sing graph convolutional neural networks to learn a representation for glycans.
Coff et al. (2020)	Regression Classifier	Glycosylation	frequent subtree mining and mRMR used for feature selection then train on regression classifier for glycan motifs.
Desaire, Patabandige & Hua (2021)	SVM	Glycosylation	The local-balanced model for improved machine learning outcomes on mass spectrometry data sets and other instrumental data
Le, Sandag & Ou (2018)	RBF Network	PTM	prediction of transport protein (including N linked) into three classes and six families using RBF Network.
Chen et al. (2021)	Deep Learning	PTM	nhKcr: a new bioinformatics tool for predicting crotonylation sites on human non histone proteins based on deep learning
Lei, Tang & Du (2017)	Ensemble Classifier	PTM	Predicting S-sulfenylation sites using physicochemical properties difference and ensemble classifier.
Murad et al. (2021)	Random Forest	PTM	Ubiquitination Sites Prediction Using Statistical Moment with Random Forest Approach.
Kumar et al. (2020)	ML	PTM	Machine Learning techniques to identify potential drug targets for Anti-epileptic drugs
Perpetuo et al. (2021)	AI	PTM	artificial intelligence be used for peptidomics
Qiu et al. (2018)	SVM	PTM	Predicting protein lysine methylation sites by incorporating single-residue structural features into Chou’s pseudo components.
Liu et al. (2021)	Extreme gradient boosting classifier	PTM	Prediction of protein ubiquitination sites *via* multi-view features based on eXtreme gradient boosting classifier.
Huang & Li (2018)	KNN	PTM	Feature extractions for computationally predicting protein post-translational modifications

It is observed from the selected articles that most of the authors (Akmal, Rasool & Khan, 2017; Taherzadeh et al., 2019; Liu et al., 2019; Lundstrøm et al., 2022; Burkholz, Quackenbush & Bojar, 2021; Kotidis & Kontoravdi, 2020; Antonakoudis et al., 2021; Hwang et al., 2020; Dimeglio et al., 2020; Dobson, Zeke & Tusnády, 2021; Ilyas et al., 2019; Chen et al., 2021) used the Artificial Neural Network (ANN) or the variant of ANN such as Deep ANN, Graph NN, Convolution NN and Recurrent NN. The second most used algorithm is Support Vector Machine (SVM) used by authors (Tran, Pham & Ou, 2021; Pitti et al., 2019; Wang et al., 2017; Desaire, Patabandige & Hua, 2021; Qiu et al., 2018) and remaining authors used Random Forest, XGBOOST, Baysen Network, Regression Classifier, Radial Base Function and some used customized method as mention in Table 11.

## Assessment of q5:

### How effective are the existing model to predict the N-Linked sites?

The result comparisons are used to present the performance to various based on which conclusion can be drawn with respect specific dimension. In this systematic review, the performance comparison of N-linked model in the selected articles has performed. The parameter used for the performance consists of (a) availability of data set. (b) accuracy metric (c) sensitivity metric. (d) specificity (e) availability of developed tool (f) comparison on independent data and the type of glycosylation as mentioned in Table 12. It is observed, most of the authors (Kotidis & Kontoravdi, 2020; Sha et al., 2019; Park et al., 2019; Antonakoudis et al., 2021; Zhang et al., 2021b; Wang et al., 2017; Kumar et al., 2020; Ilyas et al., 2019; Sugár et al., 2021; Bojar et al., 2021a; Perpetuo et al., 2021; Huang & Li, 2018) did not provide the results or they did not follow provided performance metrics in their research. The authors (Akmal, Rasool & Khan, 2017; Chien et al., 2020; Taherzadeh et al., 2019; Tran, Pham & Ou, 2021; Liu et al., 2019; Li et al., 2019; Pitti et al., 2019; Hwang et al., 2020; Le, Sandag & Ou, 2018; Dimeglio et al., 2020; Magaret et al., 2019; Ruiz-Blanco et al., 2017) mentioned most of the performance metrics specific to N-Linked sites identification and out of these, authors Chien et al. (2020) and Hwang et al. (2020) has not provide the data set on which experiments have performed.

**Table 12:** Performance comparison of N-linked models.

Ref.	Glycosylation type	Result comparison on	Tool	Dataset	ACC (%)	SN (%)	SP (%)	Finding
Akmal, Rasool & Khan (2017)	N-Linked	Yes	No	Yes	99.9	99.8	99.9	Detail Comparison has perform and also present metrics but tool is not available
Chien et al. (2020)	N-Linked	Yes	Yes	No	84.7	82.8	84.8	Detail Comparison has performed and also present metrics. But data set is not available
Taherzadeh et al. (2019)	N-Linked and O-Linked	Yes	Yes	Yes	97.5	98	–	Detail Comparison has performed and also present metrics.
Tran, Pham & Ou (2021)	N-Linked	Yes	No	Yes	93.4	98.6	92.8	Detail comparison has perform and also present metrics but tool is not available
Liu et al. (2019)	N-Linked	No	Yes	Yes	50	–	–	Not compare the result properly.
Li et al. (2019)	N-Linked (and C/O-Linked)	Yes	Yes	Yes	88.6	–	–	Detailed comparison has performed but SN and SP not computed
Bojar et al. (2021b)	Glycosylation	No	No	Yes	75	–	–	Result not compare properly and also missing few metrics.
Carpenter et al. (2022)	Glycosylation	No	Yes	No	75	–	–	Tool is available but data set is missing and did not perform all performance metric
Pitti et al. (2019)	N-Linked	Yes	Yes	Yes	74	49	–	Detailed comparison has performed but SP.
Lundstrøm et al. (2022)	Glycosylation	No	No	Yes	72	–	–	Result are not performed properly as missing metrics and tool.
Burkholz, Quackenbush & Bojar (2021)	Glycosylation	No	Yes	Yes	85	–	–	Detailed comparison performed but missing few metrics
Kotidis & Kontoravdi (2020)	N-Linked	No	No	Yes	–	–	–	Did not specify results.
Sha et al. (2019)	N-Linked	No	No	Yes	–	–	–	Did not specify results.
Park et al. (2019)	N-Linked and O-Linked	No	Yes	No	–	–	–	Did not specify results.
Antonakoudis et al. (2021)	N-Linked	No	No	Yes	–	–	–	Did not specify results.
Hwang et al. (2020)	N-Linked	No	No	No	99	100	–	Achieved almost full accuracy but result comparison with independent data set, data set and tool is missing.
Coff et al. (2020)	Glycosylation	No	Yes	Yes	89	–	–	Achieve good result but comparison on independent data set is missing and glycosylation type is not specified.
Le, Sandag & Ou (2018)	PTM (including N Linked)	Yes	Yes	Yes	92	–	–	Detailed comparison has performed and achieved good results but not specify the PTM type.
Zhang et al. (2021b)	N-Linked	Yes	No	No	–	–	–	Did not specify results.
Wang et al. (2017)	N-Linked	Yes	No	No	–	–	–	Did not specify results.
Dimeglio et al. (2020)	N-Linked	Yes	Yes	Yes	88	86	89	Detailed comparison has performed and also achieved good results.
Kumar et al. (2020)	PTM	Yes	No	Yes	–	–	–	Did not specify results.
Ilyas et al. (2019)	PTM	Yes	No	Yes	–	–	–	Did not specify results.
Magaret et al. (2019)	N-Linked	Yes	No	Yes	86	97	39	Detailed comparison has performed and also achieved good results.
Sugár et al. (2021)	N-Linked	No	Yes	Yes	–	–	–	Did not specify results.
Bojar et al. (2021a)	N-Linked	No	No	Yes	–	–	–	Did not specify results.
Desaire, Patabandige & Hua (2021)	Glycosylation	No	No	Yes	98	–	–	Achieve good result but glycosylation type is not specified and missing few metrics
Chen et al. (2021)	PTM	Yes	Yes	Yes	85	62	90	Detailed comparison has performed and achieved good result, but it is generic for PTM as specific type was not mentioned
Perpetuo et al. (2021)	PTM	No	No	No	–	–	–	Did not specify results.
Liu et al. (2021)	PTM	No	Yes	Yes	97	–	–	Detailed comparison has performed and achieved good result, but SN and SP are missing
Ruiz-Blanco et al. (2017)	N-Linked	No	No	Yes	99	82	–	Detailed comparison has performed and achieved good result, but data set is missing.
Huang & Li (2018)	PTM	No	No	Yes	–	–	–	Did not specify results.

## Discussion and future direction

This section summarizes and discuss the detail of this systematic literature review regarding the identification of N-linked sites.

## Taxonomy hierarchy

**Figure 8:**
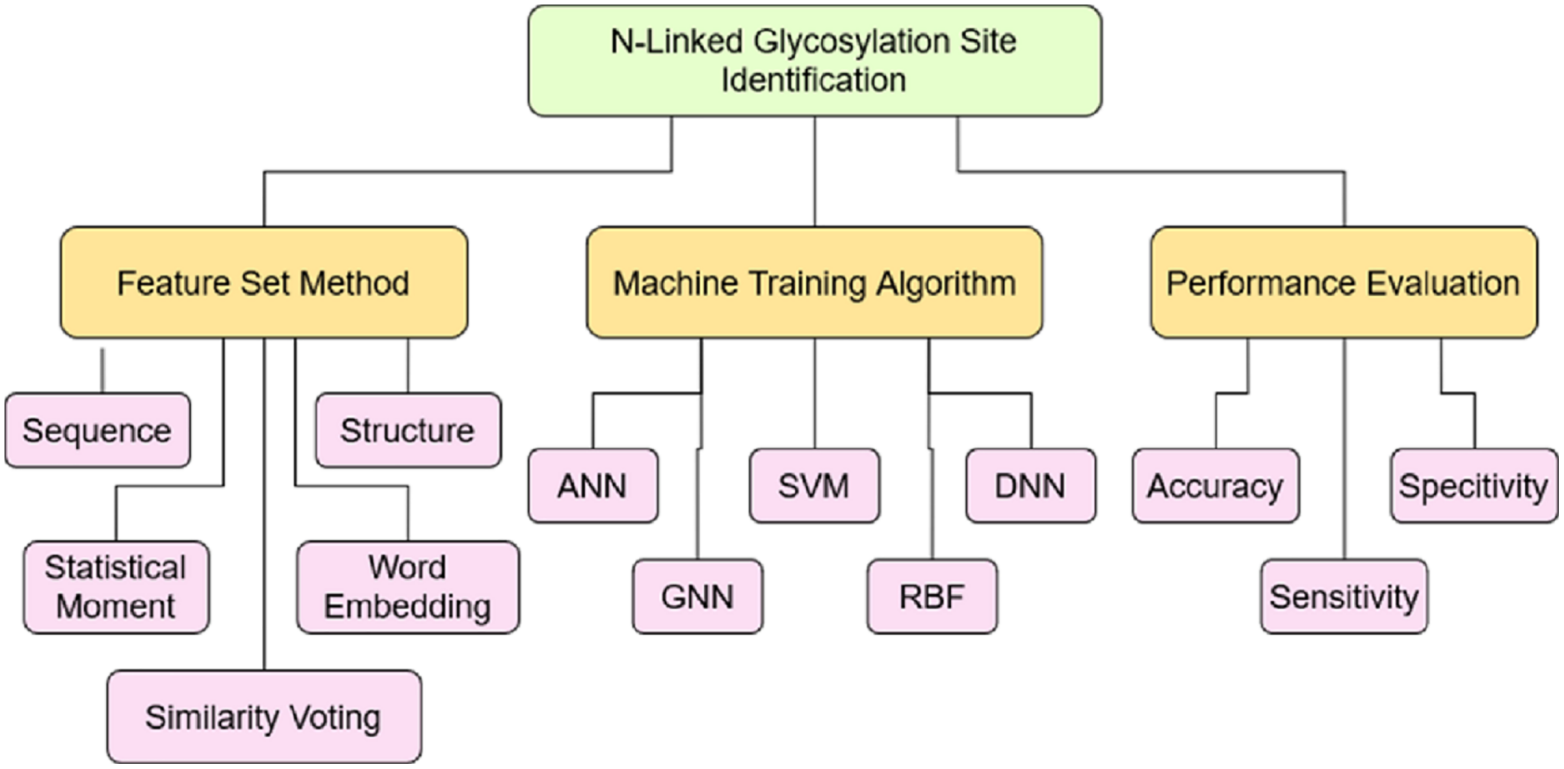
Taxonomy of N-Linked site identification perspective.

The objective of this study was to analyze the current progress to identify the N-linked glycosylation sites. To achieve this objective, a taxonomy has built based on the coding scheme as mentioned in Table 13 after critically analyzing 70 articles, selected through a systematic approach. The coding developed on the various aspects related to this study such as: Feature set construction method, machine model training algorithm and performance evaluation. These aspects are further divided into the sub-level showing the depth of each aspect and their role in the efficient identification of N-linked sites. The coding scheme helped to construct the taxonomy as shown Fig. 8 to further investigate domain and sub-domains identified through it.

**Table 13:** Taxonomy coding scheme for SLR.

Domain	Code	Subdomain	Reference
Feature set method	SMF	Statistical Moment Feature	Akmal, Rasool & Khan (2017), Le, Sandag & Ou (2018), Murad et al. (2021), Burkholz, Quackenbush & Bojar (2021), Dimeglio et al. (2020)
	SEF	Sequence Based Feature	Chien et al. (2020), Taherzadeh et al. (2019), Liu et al. (2019), Li et al. (2019), Bojar et al. (2021b), Park et al. (2019), Huang & Li (2018), Lundstrøm et al. (2022)
	SQF	Structure Based Feature	Chien et al. (2020), Taherzadeh et al. (2019), Li et al. (2019), Park et al. (2019), Huang & Li (2018), Zhang et al. (2021b), Suga, Nagae & Yamaguchi (2018), Kumar et al. (2020)
	WEF	Word Embedding Feature	Tran, Pham & Ou (2021)
	SVF	Similarity Voting Feature	Pitti et al. (2019)
Machine training algorithm	ANN	Artificial Neural Network	Akmal, Rasool & Khan (2017), Liu et al. (2019), Kotidis & Kontoravdi (2020), Antonakoudis et al. (2021), Dimeglio et al. (2020), Ilyas et al. (2019)
	SVM	Support Vector Machine	Akmal, Rasool & Khan (2017), Taherzadeh et al. (2019), Tran, Pham & Ou (2021), Desaire Patabandige & Hua (2021), Qiu et al. (2018), Pitti et al. (2019), Wang et al. (2017)
	DNN	Deep Neural Network	Taherzadeh et al. (2019), Hwang et al. (2020), Chen et al. (2021)
	GNN	Graph Neural Network	Burkholz, Quackenbush & Bojar (2021), Lundstrøm et al. (2022)
	RBF	Radial Basis Function	Le, Sandag & Ou (2018)
Performance metric	ACC	Accuracy	Akmal, Rasool & Khan (2017), Chien et al. (2020), Taherzadeh et al. (2019), Tran, Pham & Ou (2021), Liu et al. (2019), Li et al. (2019), Hwang et al. (2020), Magaret et al. (2019), Pitti et al. (2019), Dimeglio et al. (2020)
	SP	Specificity	Akmal, Rasool & Khan (2017), Chien et al. (2020), Magaret et al. (2019), Tran, Pham & Ou (2021), Dimeglio et al. (2020)
	SN	Sensitivity	Akmal, Rasool & Khan (2017), Chien et al. (2020), Taherzadeh et al. (2019), Hwang et al. (2020), Magaret et al. (2019), Ruiz-Blanco et al. (2017), Tran, Pham & Ou (2021), Pitti et al. (2019), Dimeglio et al. (2020)

## General observation and future direction

Several possible observations can be made in the finding of this SLR based on the taxonomy as shown in Fig. 8. Various RQs were developed which plays a key factor in the identification of N-linked sites. The trends and finding can be observed while the identification of such sites. These include the following observation along with future direction.

(a) **Feature set construction method** The performance of computational model deeply depends on the quality of feature set extracted from the data set which later used for training the machine learning model (Saeed, Mahmood & Khan, 2018; Khan et al., 2019; Naseer et al., 2021a). The discriminating features helps the model to learn proficiently and then perform the right prediction. Therefore, it is significant to discover the techniques which extract the useful information from the dataset. The various methods have been used by authors to construct the feature set, the widely used are: protein sequence feature, protein structure feature, statistical moments, word embedding technique and similarity voting. The majority of the authors (Liu et al., 2019; Bojar et al., 2021b; Magaret et al., 2019; Bojar et al., 2021a) only used the sequence based information of protein to train the model. It has also observed, the authors (Akmal, Rasool & Khan, 2017; Taherzadeh et al., 2019; Li et al., 2019; Park et al., 2019; Murad et al., 2021) applied the combination of multiple features such as sequence, structural and statistical to construct feature vector. More than 50% of the research article selected in this study, which got 10 points based on quality assessment score used combination of various features as mentioned above. The new techniques adopted in recent research articles are word embedding vector, graph statistical feature along with similarity voting and Chou’s five step method. The researchers can use these feature extraction techniques to improve the performance of N-linked prediction model or any PTM site identification model.
(b) **Machine training algorithm** The most significant part of computational model after the feature extraction method is to develop the method to train the machine model (Hussain, Rasool & Khan, 2020; Barukab et al., 2022; Khan et al., 2020a). The performance of model impacted most by the technique used for training the machine. The appropriate learning algorithm along with fine feature extraction method, results highly adequate model that predicts the independent data with great accuracy. Therefore, the development of appropriate machine learning method is very much essential. The researchers proposed various methods to predict the N-linked sites accurately. The most widely used methods include: Artificial Neural Network (ANN), Support Vector Machine (SVM), Deep Neural Network (DNN), Graph Neural Network (GNN) and Radial Basis Function (RBF) Network. The research article published in Q1 journal according to the JCR, used the ANN (Akmal, Rasool & Khan, 2017; Liu et al., 2019; Dimeglio et al., 2020) widely along with SVM (Taherzadeh et al., 2019; Pitti et al., 2019) method. It has also been analysed the research article (Taherzadeh et al., 2019; Le, Sandag & Ou, 2018; Ruiz-Blanco et al., 2017) in which web server has provided and present the accuracy above 90% used the Jrip Classifier, DNN, SVM and RBF algorithm. The authors (Akmal, Rasool & Khan, 2017; Tran, Pham & Ou, 2021; Hwang et al., 2020; Magaret et al., 2019; Desaire, Patabandige & Hua, 2021) who proposed prediction model without providing the webserver and also have accuracy above 90% used ANN, SVM, DNN and RF algorithms. The researchers can use these algorithms to improve the performance of N-linked prediction model or any PTM site identification model.
(c) **Performance evaluation** Once the model has trained, it then validated on the independent data to evaluate the performance. There are various techniques to measure the validity of model, the most significant metrics to evaluate the performance are Accuracy metric, Sensitivity and Specificity metric. The sensitivity test measures the true positive accuracy of a model while specificity measures the true negative accuracy of the model. In this study, the performance has evaluated on aforementioned metrics. Around 40% of the authors have not validated their model on any of above mentioned performance metrics. Only 20% of the authors have performed each of the defined performance metrics. The predictive models in which PTM type is specialized to N-linked have better accuracy as compared to those in which PTM type is not specified or are the generalized ones. The highest accuracy of −99% was achieved by author Akmal, Rasool & Khan (2017) based on these evaluation criteria. It also presents the sensitivity and specificity measures of the model which were 99.8% and 99.9% respectively, but it did not provide the web server. The author Hwang et al. (2020) claims the accuracy of 99% along with the sensitivity of 100%, but did not provide the working tool, dataset, and result comparisons with other predictors. The most efficient predictive models with available web server are Sequon model Ruiz-Blanco et al. (2017) and Sprint-Gly model Taherzadeh et al. (2019) with the accuracy of 97.5% and 97% respectively. The Sequon model has trained on the human protein sequence only while Sprint-Gly is equally effective for both human and rat species. Therefore, Sprint-Gly considered to be a reliable model out of the currently available web servers.

### Future direction

Bioinformatics is an emerging filed, there are lot of problems that needs the computational solution over the experimental. As it was mentioned earlier, the researchers have identified almost ∼ 200 types of PTM which plays key role in various biological functions. Apart from N-linked glycosylation, the other types of glycosylation such as O-linked and C-linked also play vital role in protein functioning and various drug discovery techniques. Therefore, it is the opportunity for the researchers, pharmaceutical and academia to develop the efficient computational model to solve the problem that needs better computational solution. Few of the existing problem that needs to be addressed are given below

(a) Identify the O-linked glycosylation sites for threonine and serine using ANN.
(b) How the performance of C-linked glycosylation can be enhanced through exiting neural network classifiers.
(c) Develop a comprehensive predictive model to classify the type of glycosylation.
(d) How effective are the exiting classifier to predict the other PTM sites?

## Conclusion

The significance of N-linked glycosylation promotes the discovery of such sites using computational methods instead of experimental method due to its limitations. In this systematic study, existing information to identify such sites was studied which covered the possible challenges and their solutions through systematic method. The research articles, related to the keywords associated with N-linked glycosylation were evaluated through five major digital libraries. In the result of search query applied to digital libraries, more than 800 articles have found and after filtering process 70 article were remained for further analysis. The results show that approximately 75% of the articles were published in recognized journals and rest belong to top conferences. It was observed that more than 40% of articles were published in the American journal followed by the Middle East with 20%. Most of the selected studies focused on the feature construction method and training algorithm, but less focused on the performance evaluation criteria and development of tool or web server.

The major shortcomings of any SLR primarily are related to search strategy, poor classification, and inaccurate data extraction. In this SLR, these deficiencies were overcome by applying the search query on five major digital libraries to reduce biasness. The results of search queries were then filtered through well-defined inclusion/exclusion criteria.

## Additional Information and Declarations

### Data Availability

The following information was supplied regarding data availability: This is a literature review and does not have raw data.
